# Association of energy-adjusted dietary inflammatory index with risk of cholelithiasis: a prospective cohort study

**DOI:** 10.3389/fpubh.2026.1858498

**Published:** 2026-07-08

**Authors:** Jiayue Zhu, Yinghong Zhai, Lei Yuan, Suming Dai, Fangyuan Hu, Xu Liu

**Affiliations:** 1 Faculty of Military Health Service, Naval Medical University, Shanghai, China; 2 Clinical Research Unit, School of Medicine, Shanghai Ninth People's Hospital Affiliated to Shanghai JiaoTong University, Shanghai, China

**Keywords:** cholelithiasis, cohort study, Cox regression models, energy-adjusted dietary inflammatory index, UK Biobank

## Abstract

**Background:**

Cholelithiasis is a common, costly gastrointestinal disease with a rising global prevalence, and diet-related inflammation is closely associated with the onset of cholelithiasis. The energy-adjusted dietary inflammatory index (E-DII) provides a robust measure of dietary inflammatory potential by accounting for total energy intake, but prospective data on its link to cholelithiasis in general populations remain scarce.

**Methods:**

We explored this association in a large prospective cohort of 202,129 adults aged 40–69 from the UK Biobank. E-DII scores were derived from 24-h dietary recall data, and Cox regression models were used to examine associations between E-DII quartiles and cholelithiasis. Restricted cubic spline (RCS) regression characterized nonlinear relationships, with subgroup and sensitivity analyses (competing risk models, propensity score matching) validating findings.

**Results:**

In fully adjusted model, the hazard ratios (HRs) with 95% confidence intervals (CIs) for cholelithiasis across higher E-DII quartiles were 1.05(0.97, 1.12), 1.00 (0.93, 1.08) and 1.19 (1.11, 1.28) compared to the lowest quartile, respectively. The RCS analysis showed a nonlinear and positive association between E-DII and cholelithiasis. Risk increased when E-DII was below −1.891, decreased slightly from −1.891 to −0.507, and increased continuously after −0.507. Subgroup and sensitivity analyses largely corroborated the primary findings, indicating robustness of the observed associations. No association was observed between E-DII and cholelithiasis-related mortality.

**Conclusion:**

In this large prospective cohort study, higher E-DII scores were associated with an increased risk of cholelithiasis, exhibiting a nonlinear pattern. These findings suggest that dietary adjustment may be beneficial for cholelithiasis prevention and warrant further targeted research.

## 1 Introduction

Cholelithiasis, defined by the formation of gallstones within the gallbladder, is a common and costly condition affecting an estimated 10–20% of adults worldwide (1, 2) and approximately 20% of the European population (3). Among all gastrointestinal conditions, it accounts for the greatest socioeconomic burden (4) and the highest rates of hospital admission (3). The prevalence of cholelithiasis continues to rise globally, driven by shifts in dietary habits, reduced physical activity, and population aging, underscoring the need for effective primary prevention strategies.

Diet ranks among the most important modifiable risk factors for cholelithiasis. It is currently accepted that diet serves as a key modulator of systemic inflammation. Inflammation not only accelerates the development and progression of cholelithiasis but also diminishes the efficacy of its treatment (5, 6). Specifically, pro-inflammatory foods, such as red meat, high-sugar foods, and foods high in trans fatty acids, promote the development of gallstones by triggering chronic inflammatory responses in the body. Certain anti-inflammatory foods, such as vegetables and nuts, help mitigate the occurrence and progression of gallstones by reducing systemic inflammation levels (7). To quantify the inflammatory capacity of diet more comprehensively, the Dietary Inflammatory Index (DII) was developed as a standardized tool to evaluate the inflammatory potential of individual diets (8, 9).

DII has gained widespread use in studies evaluating the effect of diet-induced inflammation on various health disorders such as malnutrition (10), maternal and infant health (11), cardiometabolic diseases (12) and cancer (13). Focusing on cholelithiasis, several epidemiological studies have reached consistent conclusions. These include a large cross-sectional analysis using 2003–2020 NHANES data (14), a BMI-matched case–control study in Iranian women (7), and cross-sectional findings from the Dena PERSIAN cohort. All these studies indicate that higher dietary inflammatory index (DII) scores are linked to an increased risk of gallstones. However, a limitation arises from the interdependence between dietary energy intake and inflammatory potential. As energy requirements and nutrient metabolism vary significantly across populations and are influenced by age and lifestyle factors (8), the Energy-Adjusted Dietary Inflammatory Index (E-DII) was subsequently introduced. By normalizing for total energy intake, the E-DII provides a more robust and physiologically relevant measure of dietary inflammation compared to the conventional DII (15).

The association between diet and the risk of cholelithiasis has attracted growing research attention in recent years. Although prior studies have explored individual dietary components and gallstone formation, systematic prospective evidence on the overall inflammatory potential of diet remains lacking, particularly using the E-DII which accounts for total energy intake. Based on these considerations, the present study investigates the prospective association between E-DII and cholelithiasis in a large general population sample.

## 2 Materials and methods

### 2.1 Study design and population

The UK Biobank^1^ is the largest repository in the UK, containing data on genes, behavioral, and environmental factors related to diseases. Its goal is to investigate the relationship between specific genes, lifestyle factors, and health outcomes. More than 500,000 participants aged 40–69 years were recruited from 2006 to 2010 and attended one of 22 assessment centers (16). With their consent, their blood, urine and saliva were regularly sampled, along with detailed information about their lifestyle, which is then linked to their health-related records to provide more insight into the individual’s experience of illness. This study was conducted in compliance with ethical standards, having received approval from the research ethics committee (11/NW/0382) and obtaining written informed consent from all participants. The data were accessed through UK Biobank under approved application number 99709.

**Figure 1:**
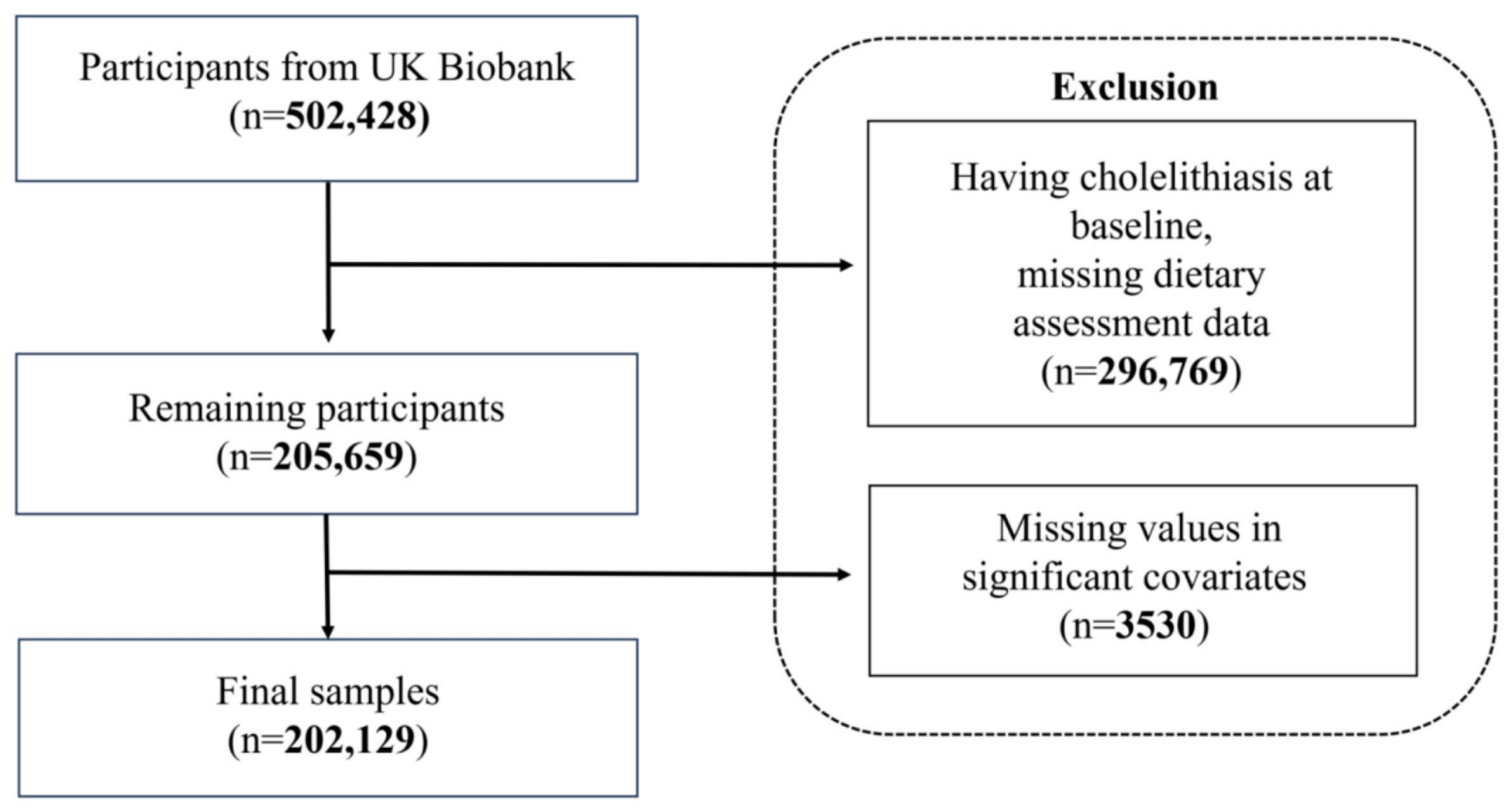
Flow diagram.

Cholelithiasis was ascertained through hospital inpatient and outpatient records using ICD-10 code K80. X, covering multiple subtypes: K80.0 (gallbladder stone with acute cholecystitis), K80.1 (gallbladder stone with chronic cholecystitis), K80.2 (uncomplicated gallbladder stone), K80.3 (common bile duct stone with cholangitis), K80.4 (common bile duct stone with cholecystitis), K80.5 (uncomplicated common bile duct stone), K80.6 (multiple intra- and extrahepatic biliary stones), K80.8 (other specified cholelithiasis), and K80.9 (unspecified cholelithiasis). Self-reported symptoms and surgical procedure codes were not used in outcome ascertainment, and gallbladder surgery status was not applied as an inclusion or exclusion criterion. Participants with any ICD-10 K80 diagnosis recorded at or prior to baseline were excluded. The data screening process is shown in Figure 1. In this prospective cohort study, we used June 1, 2023 as the cutoff date for cohort data collection. The median follow-up duration was 12.82 years (IQR: 9.22–14.54).

### 2.2 Assessment of energy-adjusted dietary inflammatory index

Dietary intake data were collected using the Oxford WebQ 24-h dietary recall questionnaire, which recorded consumption of 206 food items and 32 beverage types. To reduce measurement error, we calculated the average of all available 24-h recalls (up to a maximum of five); the use of averaged repeated measurements has been validated to improve dietary intake estimation (17). Assessments were conducted between April 2009 and June 2012.

To calculate the E-DII score, all reported intakes of dietary parameters were converted to intake per 1,000 kcal of energy. The energy-adjusted scores were computed using the mean and standard deviation of each dietary parameter, with all values standardized to per 1,000 kcal of energy (18). In this study, due to data availability, only 26 out of the original 45 validated DII dietary components were obtainable from UK Biobank, and all selected nutrients are listed in Table 1 for E-DII calculation. Mean values from UK Biobank and these 26 dietary parameters were used for E-DII calculation in this study. These parameters were used to derive z-scores relative to the population mean, which were then converted to proportions ranging from 0 to 1 and centered using the formula: 2 × proportion − 1 (19). For each dietary parameter, the centered score was multiplied by its corresponding inflammatory weight to generate a parameter-specific DII score. Higher E-DII values represent a more pro-inflammatory dietary profile. The E-DII quantifies the inflammatory potential of macronutrients, micronutrients, and other dietary components as a comprehensive measure of diet-induced inflammatory capacity (7).

**Table 1:** Nutrients in quartiles of E-DII in UK Biobank.

Nutrients	Quartile of E-DII, Median (IQR)			
	Q1	Q2	Q3	Q4
Alcohol	7.47 (20.49)	9.42 (25.67)	12.19 (31.02)	8.53 (32.50)
Vitamin B12	5.98 (4.04)	5.77 (3.47)	5.59 (3.19)	5.18 (3.07)
Vitamin B6	2.16 (0.80)	2.07 (0.79)	1.97 (0.81)	1.74 (0.86)
Vitamin C	164.15 (98.26)	129.63 (83.45)	103.02 (75.65)	70.96 (67.27)
Vitamin D	3.28 (3.99)	3.00 (3.07)	2.85 (2.65)	2.50 (2.27)
Vitamin E	10.90 (5.29)	10.65 (5.33)	10.38 (5.57)	9.45 (5.93)
Vitamin A	1115.58 (777.36)	756.77 (624.96)	649.45 (499.54)	571.05 (431.47)
Beta carotene	4332.75 (3412.29)	2246.65 (2397.25)	1456.48 (1790.35)	859.68 (1144.53)
Carbohydrate	234.76 (87.94)	247.22 (89.67)	252.48 (95.68)	257.27 (109.18)
Cholesterol	182.25 (145.50)	203.26 (153.32)	218.57 (161.10)	223.44 (173.56)
Total fat	59.08 (28.34)	68.14 (31.06)	74.30 (34.55)	79.93 (40.50)
Fiber	20.75 (8.30)	18.18 (7.22)	16.25 (6.92)	13.72 (6.94)
Iron	12.57 (4.69)	12.33 (4.56)	12.03 (4.62)	11.22 (4.97)
Magnesium	347.92 (115.55)	334.78 (109.36)	320.37 (109.20)	293.02 (115.15)
Niacin	37.77 (13.28)	37.58 (13.33)	37.40 (13.95)	35.34 (15.32)
Zinc	9.34 (3.78)	9.45 (3.84)	9.49 (4.02)	9.17 (4.53)
Omega 3	1.95 (1.33)	1.85 (1.15)	1.80 (1.09)	1.63 (1.07)
Omega 6	9.64 (5.79)	10.36 (5.94)	10.56 (6.10)	9.90 (6.22)
Protein	78.36 (26.77)	79.07 (27.22)	79.62 (28.52)	77.43 (32.38)
MUFA	21.49 (11.17)	24.64 (12.09)	26.77 (13.22)	28.14 (15.21)
Riboflavin	1.89 (0.75)	1.89 (0.74)	1.87 (0.76)	1.76 (0.83)
Saturated fat	19.57 (10.58)	24.25 (12.17)	27.71 (13.99)	32.29 (17.46)
Selenium	51.91 (30.36)	49.37 (27.50)	47.75 (25.90)	43.80 (24.49)
Thiamin	1.93 (0.83)	1.84 (0.80)	1.73 (0.78)	1.52 (0.80)
Trans fatty acids	0.82 (0.62)	1.04 (0.69)	1.19 (0.76)	1.36 (0.93)
Green tea	443.33 (570.00)	418.00 (522.50)	380.00 (601.67)	190.00 (570.00)

MUFA, monounsaturated fatty acids.

### 2.3 Covariates

We included multiple factors as covariates to account for potential confounders that have been validated in previous studies: sex (female or male), age at recruitment (years), BMI (calculated as weight (kg) divided by height (m) squared), ethnic background (categorized as White, African, Asian, mixed and other ethnic groups), university education (yes or no), alcohol drinker status and smoking status (never, previous, or current), diabetes, hypertension, dental problems and cancer were all answered by yes or no.

### 2.4 Statistical analysis

Baseline characteristics were expressed as percentage, and were compared by people without cholelithiasis (195988) and those with cholelithiasis (6141) using Chi-square test and Cochran–Mantel–Haenszel test. The E-DII in the UK Biobank was presented in quartiles and the content of each nutrient expressed as the median.

Associations between E-DII and cholelithiasis were investigated using Cox proportional hazards models. The following three models were constructed by adjusting the covariates: (1) Model 1 was adjusted for demographic variables including sex, age at recruitment, ethnic background and BMI; (2) Model 2 was adjusted for variables in Model 1 plus common disease variables like dental problems, diabetes, hypertension and cancer; (3) Model 3 was additionally adjusted for behavioral factor variables (Alcohol drinking status, Smoking status and University education) based on Model 2. The *p*-values for the cox-regression models were calculated for each model, and hazard ratios (HRs) were obtained for Q2, Q3, and Q4 using Q1 as a reference, along with their 95% confidence intervals (95% CIs). Restricted cubic spline regression was used to test the nonlinear correlation between E-DII and cholelithiasis incidence in total population and different subgroups based on model3 (population grouped by sex, age, hypertension, diabetes and dental problems). The number of knots was determined by sample size, with 5 knots specified for the overall analysis; two inflection points were identified at E-DII values of −1.891 and −0.507. No clinically predefined reference value was imposed, as E-DII does not carry an established clinical threshold, and the reference was therefore assigned automatically by the modeling software. All RCS models were adjusted for the same covariates as Model 3. Overall associations were estimated using Cox proportional hazards regression, and departure from linearity was evaluated with the likelihood ratio test. HRs and 95% CIs were shown across subgroups to further explore the potential modifiers of the association between E-DII and cholelithiasis.

For cholelithiasis-related mortality, we fitted Cox proportional hazards models after excluding participants with pre-existing cholelithiasis at baseline. The analysis therefore included all 202,129 participants free of cholelithiasis at baseline, and the outcome was death with cholelithiasis (ICD-10 K80) as the underlying cause.

Furthermore, we performed sensitivity analyses to examine the robustness of the associations. For cholelithiasis incidence, a competing-risk regression model was applied, with all-cause death regarded as the competing event, to adjust for potential bias caused by death prior to the occurrence of cholelithiasis. For cholelithiasis-related mortality, propensity score matching (PSM) was carried out to balance baseline confounding factors, and the covariates used for matching were completely consistent with those adjusted in Model 3. We performed 1:1 nearest neighbor matching without replacement, with a caliper width of 0.1. Covariate balance before and after matching was evaluated using standardized mean differences (SMD). All statistical analyses and graph plotting were performed using SAS (version 9.4) and R (version 4.1.3). Two-sided statistical tests were performed with *α* = 0.05, *p*-values < 0.05 were considered statistically significant.

## 3 Results

### 3.1 Baseline characteristics

In this cohort, the study population included 195,988 participants free of cholelithiasis and 6,141 individuals diagnosed with cholelithiasis. White participants constituted the predominant ethnic group in both subgroups, accounting for 95.75% in the non-cholelithiasis group and 97.20% in the cholelithiasis group. In general, most subjects from the two groups were unaffected by prevalent chronic disorders including diabetes and hypertension. Detailed demographic and clinical baseline characteristics of all participants are listed in Table 2.

**Table 2:** Characteristics of study population in UK Biobank.

Characteristics	Non-Cholelithiasis group (195988) *n* (%)	Cholelithiasis group (6141) *n* (%)	*p*-value	Methods
Sex			<0.0001	Chisq
Male	89,593 (45.71)	2,301 (37.47)		
Female	106,395 (54.29)	3,840 (62.53)		
Age at recruitment			<0.0001	Chisq
<60	117,798 (60.10)	3,261 (53.10)		
≥60	78,190 (39.90)	2,880 (46.90)		
BMI(kg/m^2^)			<0.0001	CMH
<18.5	1,601 (0.82)	51 (0.83)		
18.5–20	4,274 (2.18)	35 (0.57)		
20–25	69,720 (35.57)	1,213 (19.75)		
25–30	81,215 (41.44)	2,570 (41.85)		
≥30	39,178 (19.99)	2,272 (37.00)		
Ethnic background			<0.0001	CMH
White	187,660 (95.75)	5,969 (97.20)		
African	986 (0.50)	11 (0.18)		
Asian	3,258 (1.66)	70 (1.14)		
Mixed	1,192 (0.61)	21 (0.34)		
Other ethnic group	2,892 (1.48)	70 (1.14)		
University education			<0.0001	Chisq
No	110,992 (56.63)	4,109 (66.91)		
Yes	84,996 (43.37)	2032 (33.09)		
Alcohol drinker status			<0.0001	Chisq
Current	184,104 (93.94)	5,623 (91.56)		
Previous	5,740 (2.93)	262 (4.27)		
Never	6,144 (3.13)	256 (4.17)		
Smoking status			<0.0001	Chisq
Current	15,362 (7.84)	501 (8.16)		
Previous	69,387 (35.40)	2,385 (38.84)		
Never	111,239 (56.76)	3,255 (53.00)		
Diabetes			<0.0001	Chisq
No	181,965 (92.84)	5,313 (86.52)		
Yes	14,023 (7.16)	828 (13.48)		
Hypertension			<0.0001	Chisq
No	129,688 (66.17)	3,115 (50.72)		
Yes	66,300 (33.83)	3,026 (49.28)		
Dental problems				Chisq
No	126,328 (64.46)	3,571 (58.15)	<0.0001	
Yes	69,660 (35.54)	2,570 (41.85)		
Cancer				Chisq
No	152,208 (77.66)	4,330 (70.51)	<0.0001	
Yes	43,780 (22.34)	1811 (29.49)		

Divided by the E-DII quartiles, Vitamin B12, Vitamin B6, Vitamin C, Vitamin D, Vitamin E, Vitamin A, beta carotene, fiber, iron, magnesium, niacin, Omega 3, protein, riboflavin, selenium, thiamin and green tea showed a decreasing trend with increasing E-DII which means they are more anti-inflammatory, whereas carbohydrate, cholesterol, total fat, MUFA, saturated fat and trans fatty acids had a positive relationship with E-DII (Table 1).

### 3.2 Association between E-DII and cholelithiasis

As shown in Table 3, E-DII was positively associated with cholelithiasis risk. When E-DII reached Q4, statistical outcomes were significant, Model 1 (HR = 1.23, 95%CI = 1.15–1.32), Model 2 (HR = 1.22, 95%CI = 1.13–1.31), and Model 3 (HR = 1.19, 95%CI = 1.11–1.28). To ensure the results were reliable, we tested E-DII as a continuous variable and found again that E-DII was positively associated with cholelithiasis risk (*p* < 0.0001), Model 1 (HR = 1.05, 95%CI = 1.04–1.07), Model 2 (HR = 1.05, 95%CI = 1.03–1.06), Model 3 (HR = 1.04, 95%CI = 1.03–1.06). Sensitivity analyses using the competing risk model further validated the robustness of our primary findings. The model confirmed that higher E-DII was significantly associated with increased cholelithiasis risk (HR = 1.051, 95% CI: 1.027–1.075, *p* < 0.001), with detailed results presented in Supplementary Table S2.

**Table 3:** Associations between E-DII and cholelithiasis incidence in the UK Biobank.

Model	Quartile of E-DII, HR (95% CI)				P_Cox-regression_	P_Competing-Risks_	Continuous E-DII, HR (95% CI)	P_Cox-regression_
	Q1	Q2	Q3	Q4				
Model 1	1 (ref)	1.04 (0.97, 1.12)	1.01 (0.94, 1.09)	1.23 (1.15, 1.32)	<0.0001	<0.0001	1.05 (1.04, 1.07)	<0.0001
Model 2	1 (ref)	1.04 (0.97, 1.12)	1.01 (0.94, 1.09)	1.22 (1.13, 1.31)	<0.0001	<0.0001	1.05 (1.03, 1.06)	<0.0001
Model 3	1 (ref)	1.05 (0.97, 1.12)	1.00 (0.93, 1.08)	1.19 (1.11, 1.28)	<0.0001	<0.0001	1.04 (1.03, 1.06)	<0.0001

Model 1: Sex, age at recruitment, ethnic background, BMI.

Model 2: Variables in Model 1 plus dental problems, diabetes, hypertension and cancer.

Model 3: Variables in Model 2 plus alcohol drinker status, smoking status and university education.

**Figure 2:**
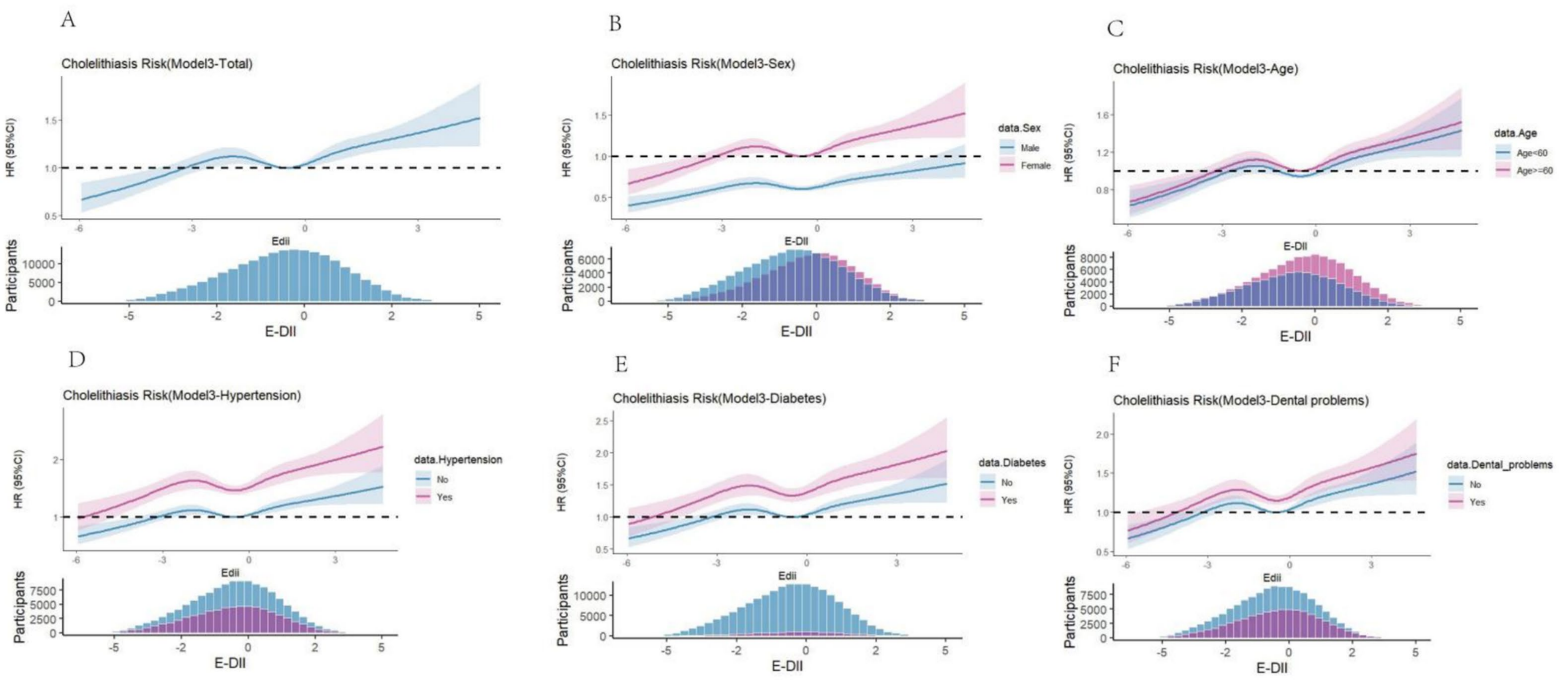
Restricted cubic spline for testing the hypothesis of nonlinear correlation between E-DII and cholelithiasis incidence, with histograms depicting participant distribution across E-DII scores. **(A)** Total population, **(B)** population grouped by Sex, **(C)** population grouped by Age, **(D)** population grouped by hypertension, **(E)** population grouped by diabetes, **(F)** population grouped by dental problems. All P_non-linearity_<0.0001.

RCS regression demonstrated a nonlinear association between E-DII and cholelithiasis (Figure 2), with mild fluctuations observed along the fitted curve and inflection points located at E-DII values of −1.891 and −0.507. Risk increased below −1.891, dropped modestly between −1.891 and −0.507, and climbed steadily as E-DII exceeded −0.507. Similar nonlinear changing patterns were observed across all predefined subgroups. A higher risk of cholelithiasis was observed in women, those older than 60 years of age, and those with common illnesses (hypertension, diabetes and dental problems).

**Figure 3:**
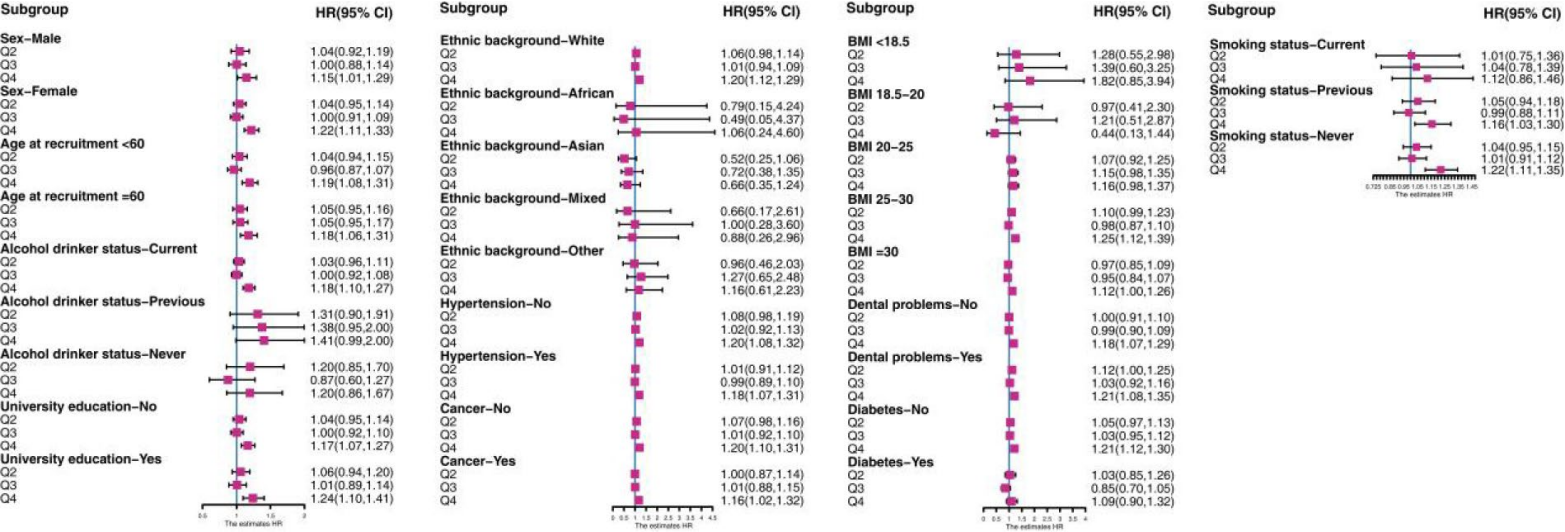
Associations between E-DII and cholelithiasis incidence across subgroups.

The results of stratified analyses were shown in Figure 3. The E-DII and cholelithiasis were positively associated across the highest quartile (Q4) in all pre-specified subgroups. These were consistent with the results observed in the primary analyses.

Cholelithiasis-related mortality represents an important clinical endpoint worth further exploration. Accordingly, we further investigated the association between E-DII and cholelithiasis-related deaths. Our analyses revealed no significant association between E-DII and cholelithiasis-related mortality, and propensity score matching confirmed consistent results (Table 4). We further used propensity score matching (PSM) as a sensitivity test to confirm result robustness. After matching, all covariates achieved adequate balance with SMD < 0.1 (Supplementary Table S1; Supplementary Figure S2), and propensity scores were well distributed across groups (Supplementary Figure S1). The PSM results were consistent with the primary analysis (Table 4).

**Table 4:** Associations between E-DII and cholelithiasis-related mortality.

Model	Quartile of E-DII, HR (95% CI)				P_Cox-regression_	Continuous E-DII, HR (95% CI)	P_Cox-regression_	P_PSM_
	Q1	Q2	Q3	Q4				
Model 1	1 (ref)	0.80 (0.64, 1.01)	0.83 (0.65, 1.04)	0.98 (0.78, 1.23)	0.8994	1.01 (0.96, 1.06)	0.6517	0.8673
Model 2	1 (ref)	0.82 (0.65, 1.04)	0.86 (0.68, 1.08)	0.95 (0.76, 1.20)	0.7804	1.01 (0.96, 1.06)	0.6428	0.5681
Model 3	1 (ref)	0.82 (0.65, 1.04)	0.84 (0.67, 1.07)	0.91 (0.72, 1.14)	0.4749	0.99 (0.95, 1.05)	0.9638	0.4230

Model 1: Sex, age at recruitment, ethnic background, BMI.

Model 2: Variables in Model 1 plus dental problems, diabetes, hypertension and cancer.

Model 3: Variables in Model 2 plus alcohol drinker status, smoking status and university education.

Cholelithiasis-related mortality was defined as death with underlying cause coded as ICD-10 K80. X.

## 4 Discussion

Among the 202,129 UKB participants included in the present study, we observed that higher E-DII was significantly associated with a higher risk of cholelithiasis. On this basis, we identified a positive nonlinear association between E-DII and cholelithiasis risk among UK general adults. Subgroup analyses further confirmed that this association remained consistent across strata defined by multiple covariates. Consistent with our results, multiple prior observational studies with different research designs have investigated the association between pro-inflammatory diet and cholelithiasis risk. For instance, Ghorbani et al. found that higher DII scores were associated with a higher risk of cholelithiasis (7); Cheng et al. revealed a significant dose–response relationship between DII and cholelithiasis (20). However, Sadri et al. demonstrated that a pro-inflammatory diet was associated with a reduced chance of developing cholelithiasis, using data from the baseline period in the Dena PERSIAN cohort (21).

Using RCS regression, we characterized the nonlinear dose–response relationship between E-DII and cholelithiasis risk, with an overall rising tendency of disease risk as E-DII increased. Luo et al. reported a “J”-shaped nonlinear association between DII and gallstone risk in a large NHANES sample, consistent with our findings, although their analysis was restricted to overweight and obese individuals (14). Biologically, pro-inflammatory dietary components elevate circulating IL-6 and TNF-*α*, which impair gallbladder epithelial absorptive function and accelerate cholesterol crystallization in bile — mechanisms supported by Mendelian randomization evidence linking circulating inflammatory cytokines causally to gallstone formation (22, 31), which helps explain the elevated cholelithiasis risk alongside higher E-DII values. Our fitted curve showed minor fluctuations within the negative E-DII range. The transient risk increase observed at lower E-DII values may be partially explained by the hypothesis from previous research that unbalanced or restrictive diets cause insufficient micronutrient intake, disturbing bile acid metabolism (21) and gut microbiota composition (23) to alter gallstone formation risk. Meanwhile, statistical factors including model smoothing and uneven sample distribution could also contribute to this subtle variation. Visual inspection of the stratified RCS curves confirmed that this non-monotonic pattern and overall curve geometry remained highly consistent across various sub-populations.

While the shape of this nonlinear relationship was shared across strata, the effect estimates derived from our Cox regression models varied significantly among specific clinical phenotypes. Subgroup analyses revealed significant associations between the E-DII and cholelithiasis among women, individuals over 60 years of age, those with higher BMI, diabetes, hypertension, and dental problems. Our findings are consistent with previous studies indicating that women are at a higher risk of gallstone disease compared to men, with factors such as pregnancy, fetal size, and obesity during pregnancy further elevating this risk (24). Chen et al. also demonstrated that each standard deviation increase in BMI, percentage body fat, and fasting insulin was associated with an increased incidence of cholelithiasis (4). Aging is another important factor associated with gallstone formation, possibly through its effects on bodily function and metabolism. Although age does not show a significant correlation with DII scores, it is notably associated with the E-DII, possibly due to reduced appetite and energy intake in older adults compared to younger populations (15). Previous cohort studies have reported a higher relative risk of cholelithiasis in individuals with type 2 diabetes than in non-diabetic groups (25), whereas alcohol consumption has been inversely associated with gallstone risk (26). Additionally, Zhang et al. observed that gallstone disease was prevalent and that hypertension was significantly associated with an increased risk, demonstrating a dose–response relationship. Their results indicated that as the severity of hypertension increased, the risk of gallstone disease rose markedly (27). Similarly, Yu et al. identified an inverted L-shaped association between missing teeth and gallstones, suggesting that appropriate lifestyle interventions in individuals with tooth loss may delay the onset of gallstones (28).

Cholelithiasis can be treated surgically (available options include laparoscopy, endoscopy, percutaneous technique, and open surgery (29)) and non-surgically (oral dissolution therapy), but there are certain complications and the treatment is expensive (30). This study suggests that cholelithiasis risk is associated with dietary inflammatory potential from a nutritional perspective, indicating a potential dietary strategy that may help reduce cholelithiasis risk. Modifying pro-inflammatory dietary patterns might be associated with a lower likelihood of gallstone disease or adverse outcomes in high-risk populations.

There are several strengths. First, this study used data from the UK Biobank, a large prospective cohort with detailed baseline measurements. This allowed us to assess E-DII before cholelithiasis developed, which clarifies the temporal association. Second, besides finding a positive relationship between E-DII and all-cause cholelithiasis, we further measured the non-linear relationship using the RCS to quantify variations in cholelithiasis risk across different E-DII ranges. Third, multiple statistical approaches including competing risk model and PSM were applied to reduce potential bias and verify result stability, improving the robustness of our findings.

Nevertheless, it is important to consider several limitations in this study. First, several potential confounders including medication use, hormone-related and reproductive indicators as well as detailed lifestyle metrics were unavailable for adjustment, which might lead to residual confounding. Second, all cholelithiasis subtypes under ICD-10 K80. X were pooled for analysis without subtype stratification due to limited available data, possibly resulting in outcome misclassification related to disease heterogeneity. Third, dietary information relied on single-time-point 24-h recall, which cannot completely reflect long-term habitual diet and carries inherent measurement error. Fourth, the UK Biobank suffers from healthy volunteer bias and predominantly consists of White participants, restricting the generalizability of our findings to other ethnic or general populations.

## 5 Conclusion

In summary, our study demonstrated a significant association of E-DII with increased risk of cholelithiasis. Dietary modification that limits pro-inflammatory foods may be associated with a reduced risk of cholelithiasis. Additional clinical trials and extended long-term follow-up are essential to validate the association between diet, inflammation and cholelithiasis and explore the biomedical mechanisms.

## Data Availability

For UKB data, public sharing is restricted. Access to these confidential data is governed by the UK Biobank Institutional Data Access/Ethics Committee (http://www.ukbiobank.ac.uk).

## Footnotes

1
http://www.ukbiobank.ac.uk
