# Supplementary material for: Association of energy-adjusted dietary inflammatory index with risk of cholelithiasis: a prospective cohort study
Source: Front Public Health. 2026 Jul 8;14:1858498. doi: 10.3389/fpubh.2026.1858498 (PMC13388382; doi:10.3389/fpubh.2026.1858498)
Supplement: Supplementary file 1 [file Table_1.DOCX]

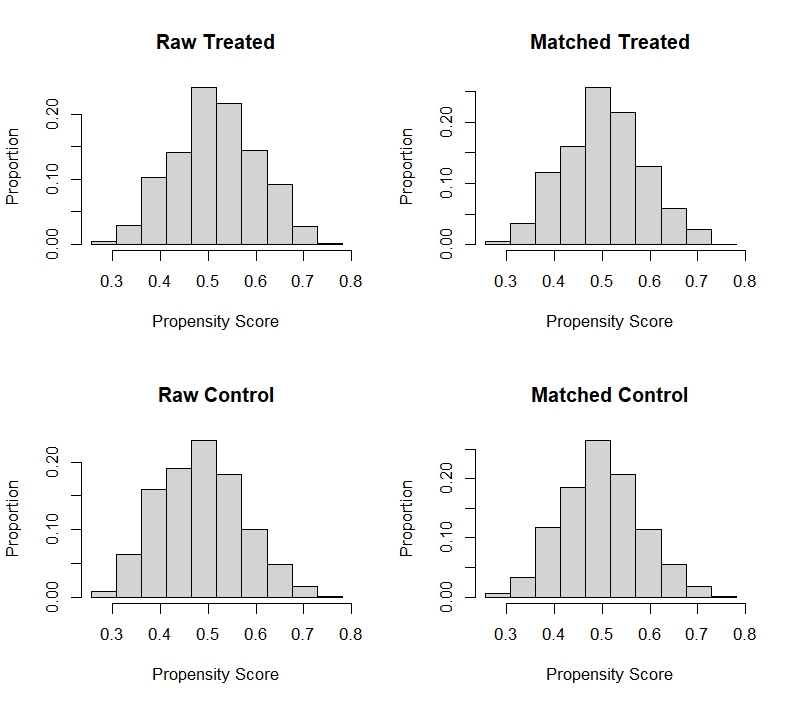


Supplementary Figure S1. Distribution of propensity scores before and after matching


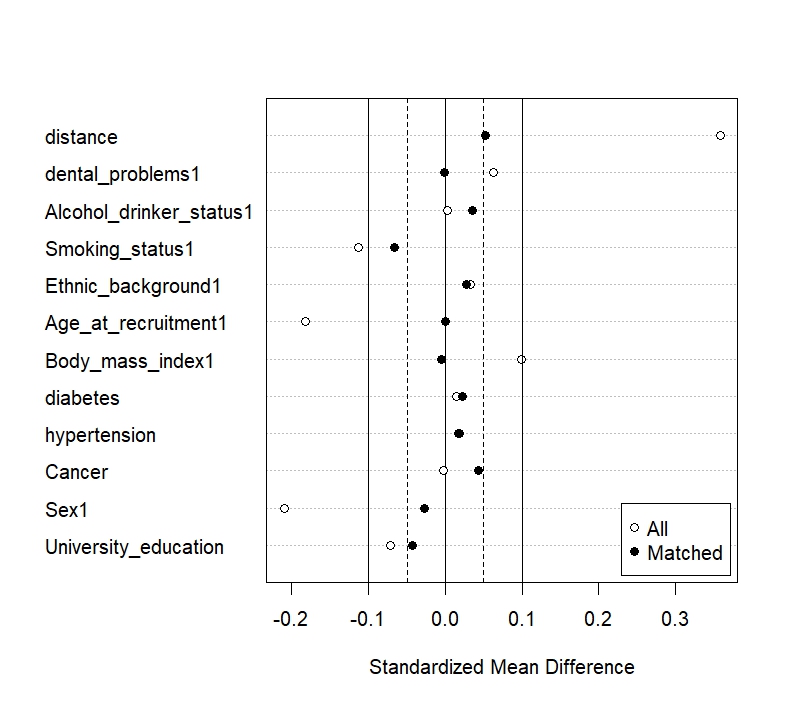


Figure S2. Standardized mean differences (SMD) of covariates before and after propensity score matching

Table S1. Standardized mean differences (SMD) of covariates before and after propensity score matching

| Variables | SMD before matching | SMD after matching |
| --- | --- | --- |
| Distance | 0.3579 | 0.0523 |
| Dental problems | 0.0628 | -0.0015 |
| Alcohol drinker status | 0.0029 | 0.0349 |
| Smoking status | -0.1125 | -0.0663 |
| Ethnic background | 0.0327 | 0.0277 |
| Age at recruitment | -0.1822 | 0 |
| Body mass index | 0.0987 | -0.0055 |
| Diabetes | 0.0150 | 0.0227 |
| Hypertension | 0.0166 | 0.0186 |
| Cancer | -0.0024 | 0.0432 |
| Sex | -0.2098 | -0.0271 |
| University education | -0.0718 | -0.0433 |

Table S2. Results of the competing risk regression model (Model 3)

| Variables | Coefficient | z-value | P-value | HR(95%CI) |
| --- | --- | --- | --- | --- |
| EDII group | 0.0496 | 4.19 | <0.001 | 1.051 (1.027, 1.075) |
| Dental problems | 0.1397 | 5.35 | <0.001 | 1.150 (1.093, 1.210) |
| Alcohol drinker status | 0.1288 | 4.44 | <0.001 | 1.137 (1.075, 1.204) |
| Smoking status | -0.0431 | -2.12 | <0.001 | 0.958 (0.920, 0.997) |
| Ethnic background | -0.1387 | -5.13 | <0.001 | 0.871 (0.826, 0.918) |
| Age at recruitment | 0.0521 | 3.83 | <0.001 | 1.053 (1.026, 1.082) |
| Body mass index | 0.4709 | 25.81 | <0.001 | 1.601 (1.545, 1.660) |
| Diabetes | 0.2741 | 6.86 | <0.001 | 1.315 (1.216, 1.422) |
| Hypertension | 0.3749 | 13.13 | <0.001 | 1.455 (1.376, 1.539) |
| Sex | 0.5147 | 18.88 | <0.001 | 1.673 (1.586, 1.765) |
| Cancer | 0.2777 | 9.69 | <0.001 | 1.320 (1.248, 1.396) |
| University education | -0.2416 | -8.73 | <0.001 | 0.785 (0.744, 0.829) |

.
